# An Investigation of the Effects of Formononetin on Hypothalamic Gonadotropin-releasing Hormone, Kisspeptin and Tachykinin 2 Gene Expression in Rats: Formononetin Affects Gnrh, Kiss1 and Tac2

**DOI:** 10.31661/gmj.v14i.3549

**Published:** 2025-02-12

**Authors:** Elaheh Basirat, Fariba Mahmoudi, Homayoun Khazali

**Affiliations:** ^1^ Faculty of Sciences, University of Mohaghegh Ardabili, Ardabil, Iran; ^2^ Department of Animal Sciences and Marine Biology, Faculty of Life Sciences and Biotechnology, Shahid Beheshti University, Tehran, Iran

**Keywords:** Formononetin, Kisspeptin, Neurokinin B, GnRH

## Abstract

**Background:**

Red clover and its main derivative, formononetin, belong to the phytoestrogens. They are clinically used to alleviate mood disorders, anxiety, and hot flashes. Formononetin may interfere with the reproductive axis due to its estrogenic potency and its ability to bind estrogen receptors. To find some molecular mechanisms mediating the effects of formononetin on the hypothalamus-pituitary-gonadal (HPG) axis, this research aimed to investigate the effects of formononetin on the hypothalamic mRNA levels of gonadotropin-releasing hormone (Gnrh), kisspeptin1(Kiss1) and tachykinin 2 (Tac2).

**Materials and Methods:**

Fifteen male Wistar rats weighing 200±10 g were divided into three groups (n=5). Group 1 as the control group, received saline. Groups 2 and 3 received 20 and 40 µg of formononetin via the third cerebral ventricle. The hypothalamic samples were dissected. The Gnrh, Kiss1 and Tac2 gene expression was measured by real-time PCR.

**Results:**

Injection of 20 µg formononetin did not significantly decrease the mRNA levels of Gnrh and Tac2 compared to the control group. However, injection of 40 µg formononetin significantly reduced the mRNA levels of Gnrh and Tac2 compared to the control group. Injection of 20 and 40 µg formononetin, significantly declined the mRNA levels of Kiss1 compared to the control group.

**Conclusion:**

Present results indicated that formononetin may be involved in the regulation of the reproductive axis via reducing the activity of hypothalamic GnRH neurons and downregulation of the kisspeptin and neurokinin B signaling pathways upstream of GnRH neurons.

## Introduction

The hypothalamic-pituitary-gonadal (HPG) axis controls reproduction [1]. Reproductive success is dependent on the cooperation of various neuropeptides and hormonal systems to regulate gonadal function and sexual behaviors. Additionally, various peripheral factors, including stress, drugs, and dietary components, can influence the HPG axis output by affecting the activation of GnRH or neurons upstream of GnRH. Stress, estrogenic drugs, and phytoestrogens could lower the release of GnRH/LH and sexual hormones [1][2][3].

The products of the Kisspeptin/ neurokinin B (NKB)/ dynorphin (KNDy) neurons have been discovered in the arcuate nucleus (ARC) of the hypothalamus [4]. The KNDy neurons form an interconnected network that regulate the GnRH. Kisspeptin directs GnRH secretion, while NKB and dynorphin act as the start and stop signals of the KNDy network, respectively [4][5]. In addition, all three peptides are thought to play separate roles in the controlling of the GnRH pulse generator.

Kisspeptin encoded by the Kiss1 gene, is expressed in the ARC and anteroventral periventricular nuclei (AVPN) of the hypothalamus. It stimulates the release of GnRH/LH [6][7]. According to previous studies the mutation of the Kiss1 or Kiss1R gene produces pubertal failure, hypogonadotropic hypogonadism, reduced gonadal size, and delayed puberty, whereas activating mutations cause early puberty [7][8].

The neurokinin B (NKB) is a decapeptide that belongs to the tachykinin family, along with neurokinin A, substance P, neuropeptide C, neuropeptide K, and hemokinin-1 [9][10]. The NKB is encoded by the Tac2 gene in rodents. The NKB is commonly expressed in the hypothalamus and other areas of the brain. It interacts with the TacR3 gene-encoded receptor named NK3R [11]. It is a significant regulator of GnRH secretion due to the interaction with kisspeptin and dynorphin signalling pathways. Its pulsatile production activates the kisspeptin release which then promotes the secretion of GnRH/LH [11].

Current chemical medicines or steroid hormone therapy have significant adverse effects, including headaches, mood changes and breast cancer. Therefore, new medicines derived from plants, as a complementary or alternative approach, are needed to reduce the adverse effects of chemical drugs. Formononetin, a phytostrogen from isoflavonoid family is the man derivative of Red clover (Trifolium pratense) [12][13]. Previous studies have shown that formononetin exhibits anti-inflammatory and antioxidant properties, which may protect against oxidative stress-related diseases [12][13][14]. It is involved in the regulation of lipid profile and blood pressure [15]. Some studies have demonstrated the role of formononetin in managing metabolic disorders like diabetes by focusing on its effects on insulin sensitivity and glucose metabolism [16]. Also, formononetin exerts anxiolytic and anticancer effects and it relieves hot flashes in menopausal women [13][17][18]. However, there is no information about the central molecular mechanism through which formononetin may affect reproductive neural pathways. Formononetin may interfere with the reproductive axis due to its estrogenic potency and its ability to bind estrogen receptors. To find some molecular mechanisms mediating the effects of formononetin on the HPG axis, this research aimed to investigate the effects of formononetin on the hypothalamic mRNA levels of Gnrh, Kiss1 and Tac2.

## Material and Methods

### Animals

A total of 15 adult male Wistar rats (200-210 g) were utilized in the present investigation. Throughout the experiment, rats were subjected to a 12/12 h light/dark cycle, having unrestricted access to food and water. All of the experiments were approved by the Research Ethics Committee of the University of Mohaghegh Ardabili (code: IR.UMA.REC.1400.028).

### Stereotaxic Surgery

By intraperitoneal injection of a combination of 10 mg/kg xylazine and 80 mg/kg ketamine, rats were anesthetized. The head of the animal was placed in the stereotaxic apparatus. The coordinates of the third cerebral ventricle were determined based on the Paxinos and Watson atlas (AP=0.84 mm, ML=00, and DV=6.5 mm) [19]. The cannula was fixed on the surface of the skull with dental cement. After one-week recovery period, the injection of drugs was done using a hamilton syringe connected to a 20 polyethylene tube and a 27 gauge needle.

### Experimental Design

Fifteen rats were divided into three groups (n=5) at random. Group 1 as control rats, received saline. Groups 2 and 3 received 20 or 40 µg formononetin via the third cerebral ventricle. The dosage of formononetin was chosen based on previous study that demonstrated the anxiolytic effects of formononetin [20].

### Real-time Polymerase Chain Reaction (RT-PCR)

The rats were sacrificed, and the hypothermic samples were extracted, frozen in liquid nitrogen, and kept at - 80˚C. Based on the acid guanidinium thiocyanate-phenol-chloroform method, TRIzol (Qiagen, Germany) reagent was used for the extraction of total RNA. The cDNA synthesis kit (Biotech rabbit, Germany) was used to convert RNA (1µg of total RNA) to cDNA. The SYBR Green (Takara Bio Inc., Japan) PCR Master Mix was then used to perform real-time PCR. The reaction was incubated at 95 Cº for 15 min, followed by 40 cycles of denaturation at 95 Cº for 20 seconds, annealing at 60 Cº for 15 sec extension at 72 Cº for 10 sec. The sequences of primers have been mentioned in the Table-1. Relative changes in mRNA levels were evaluated using the 2-ΔΔCT method. GAPDH was used to normalize the gene expression of each sample.

### Statistical Analysis

The experimental data were analyzed using SPSS software (version 16), one-way ANOVA with a post-hoc Tukey test. Statistical significance was set at P≤0.05. Mean ± SEM was used to express the results.

## Results

**Figure-1:**
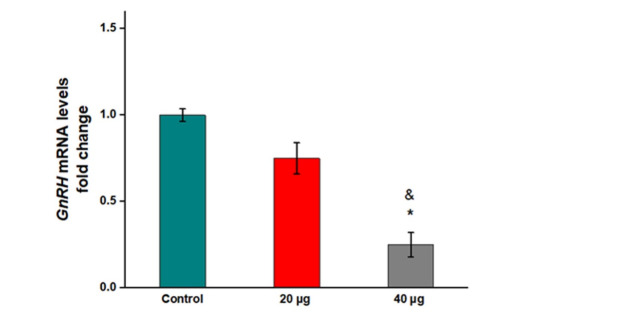
Figure 1. mpacts of formononetin on mRNA levels of Gnrh in the hypothalamus of rats. *: compared with control, &: compared with 20 µg group.

**Figure-2:**
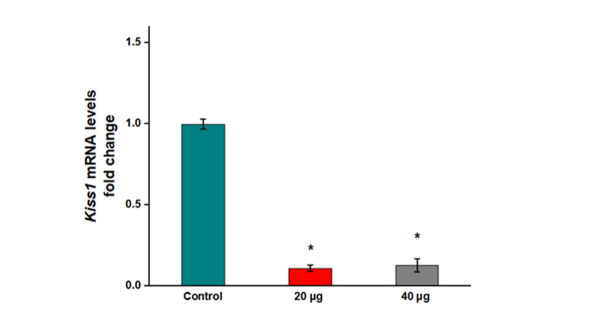
Figure 2. Impacts of formononetin on mRNA levels of Kiss1 in the hypothalamus of rats. *: compared with control.

**Figure-3:**
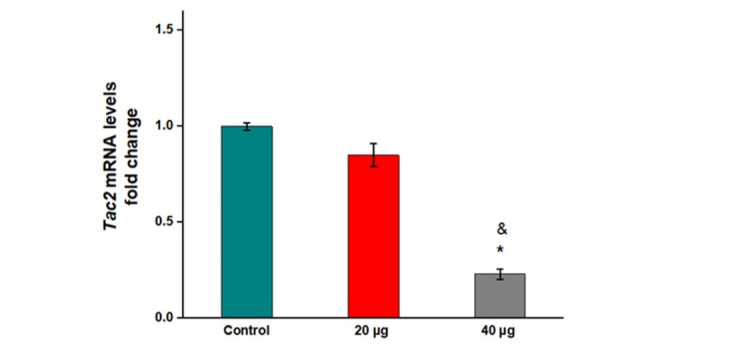
Figure 3. Impacts of formononetin on mRNA levels of Tac2 in the hypothalamus of rats. *: compared with control, &: compared with 20 µg group.

**Table:** Table1. Sequence of Forward and Reverse Primers

	primers sequences
GnRH *:*	5′- GGCTTTCACATCCAAACAGA -3′ 5′- GCCTTCCAAACACACAGTCA -3′.
Kiss1:	5′- TGATCTCGCTGGCTTCTTGGC -3′ 5′- GGGTTCAGGGTTCACCACAGG -3′.
Tac2:	5′- GGAAGGATTGCTGAAAGTGCTGAG -3′ 5′- GGGAGTGTCTGGTTGGCTGTTC -3′.
GAPDH:	5′- AAGTTCAACGGCACAGTCAAG -3′ 5′- CATACTCAGCACCAGCATCAC -3′.

Injection of 20 µg formononetin did not cause a remarkable reduction in the mRNA levels of Gnrh compared to the control group (Figure-1, P=0.151). However, 40 µg formononetin significantly reduced the mRNA levels of Gnrh in comparison to the control (Figure 1, P=0.01). Also, the result indicated a significant decrease in the mRNA levels of Gnrh in the hypothalamus of rats receiving 40 µg formononetin compared to ones that received 20 µg formononetin (Figure-1, P=0.09).

The mRNA levels of Kiss1 remarkably reduced in the animals receiving 20 and 40 µg formononetin compared to the control (Figure-2, P=0.000 and P=0.000). However, a significant reduction was not occurred between the influences of 20 and 40 µg formononetin on Kiss1 mRNA levels (Figure-2, P=0.964).The mRNA levels of Tac2 did not decline significantly in rats receiving 20 µg formononetin compared to the control (Figure-3, P=0.262). The mRNA levels of Tac2 remarkably decreased in the group of 40 µg formononetin compared to control (Figure-3, P=0.000). Also, a significant decrease occurred between the impacts of 20 and 40 µg formononetin on the mRNA levels of Tac2 (Figure-3, P=0.001).

## Discussion

Present results indicated that formononetin caused a remarkable reduction in the hypothalamic mRNA levels of Gnrh. The present findings are consistent with the previous ones which documented phytostrogens and 17 β- β-estradiol (E2) may downregulate the expression of Gnrh gene [21][22]. The E2 could participate in the regulating of reproduction by hyperpolarizing GnRH neurons and inhibiting their firing rate via activating inward rectifying K channels and blocking Na channels. E2 is able to alter GnRH neurons activity via postsynaptic binding to ERα and presynaptic binding to ERβ [22]. Several previous studies in male rodents, men and other species documented that androgens and hypothalamic estrogen derived from testosterone by the action of enzyme aromatase are involved in the negative feedback controlling GnRH/LH release. In addition to inhibiting the release of GnRH, hypothalamic estrogen inhibits the response of the pituitary gland to GnRH [23]. In fact, dysfunction of hypothalamic 17 β-estradiol could disrupt the HPG activity and natural reproduction process in males [23][24].

Phytoestrogens which are consumed to improve mood, anxiety, hot flashes, or cognitive function may interfere with the action of the reproductive axis due to their estrogenic potency and their interference with the estrogen receptors [3][25]. Formononetin exhibits direct binding potency to both estrogenic receptor (ER) subtypes, ERα and ERβ. So, formononetin may have the ability to trigger some functions evoked by the estrogens [26][27]. Both Red clover and formononetin are capable of increasing serum concentration of E2 in menopausal women and PCOS patients [18][28][29]. So, the estrogenic potency of formononetin may be a possible mechanism to inhibit the hypothalamic Gnrh.

To unravel some intra-hypothalamic mechanisms which through formononetin may inhibit the GnRH, the present research aimed to study the alteration of kisspeptin and NKB circuits activity upstream of the GnRH neurons. As expected, formononetin inhibits hypothalamic Kiss1 and Tac2 gene expression. As previous studies demonstrated disturbance of kisspeptin/GPR54 and NKB signaling pathway is a crucial factor in the physiopathology of reproductive disorders, and over-secretion of kisspeptin and NKB is linked to the overproduction of GnRH [4]. Also, in addition to the co-expression of NKB and dynorphin for driving GnRH/LH pulses, kisspeptin neurons of the ARC nucleus co-express glutamate and glutamate transporter [30][31]. In gonadectomized males, the expression of gene-coding glutamate transporter and glutamate release are elevated in the kisspeptin neurons which is a demonstration of the suppressive impact of gonadal steroids on glutamate in these neurons [31][32]. In addition, it is documented that E2 treatment leads to decrease in the number of kisspeptin and NKB neurons and it inhibits the Kiss1 and Tac2 mRNA expression in the ARC nucleus which is responsible for the tonic pulsatile release of GnRH/LH in both sexes [30][32]. In fact, the synchronized activity of kisspeptin neurons of ARC is essential to trigger the pulsatile GnRH secretion [33]. It has been revealed that glutamate induces the synchronous activity of kisspeptin neurons and NKB potentiates the glutamate-driven synchronizations in the KNDy neural circuits to control GnRH release [33].

Based on several studies, the physiological activity of formononetin is linked to the downregulation of the glutamatergic signaling pathway. Formononetin is capable of suppressing the gene expression of glutamate receptors [17]. Also, analgesic impacts of formononetin have been shown in a rat model of glutamate-induced nociception [34][35]. Studying the neuroprotective potency of formononetin, established its suppressive effects against glutamate-induced cell death [35][36]. So, downregulation of the glutamatergic signaling pathway could be a possible mechanism that through formononetin may decline the mRNA levels of Kiss1 and Tac2.

The potential therapeutic implications of the present findings may be helpful to the damping of menopausal hot flashes which are correlated with a significant decrease in steroid hormones and higher secretion of GnRH/ LH [37]. Over secretion of GnRH/LH is associated with the overproduction of kisspeptin and NKB upstream GnRH neurons [4] and menopausal women suffer from elevated levels of GnRH, kisspeptin and NKB [4][10][38]. It has been demonstrated that kisspeptin and NKB signaling pathways are important mediators for the induction of menopausal hot flushes and they link estrogen deficiency to hot flushes [4]. As, the present results demonstrated the inhibitory effects of formononetin on mRNA levels of Gnrh, Kiss1, and Tac2. So, formononetin may be supposed to have clinically important therapeutic implications for reducing hot flashes due to its association with the blockade of NKB and kisspeptin signaling pathways.

## Conclusion

The results indicated that a third cerebral ventricle injection of formononetin significantly reduced the mRNA levels of Gnrh in the hypothalamus of male rats. This finding implies a direct link between the downregulation of kisspeptin and NKB signaling pathways and the reduction of the mRNA levels of hypothalamic Kiss1 and Tac2 in the formononetin-treated rats. The present study highlights formononetin’s potential involvement in controlling the HPG axis. However, further studies are needed to investigate the role of formononetin in the regulation of other intra-hypothalamic reproductive signaling pathways upstream GnRH neurons such as ghrelin, neuropeptide Y, leptin, orexin and corticotrophin-releasing hormone (CRH) in intact or ovarian polycystic models of rats. One important limitation of the present study could be the impossibility of using the western blot technique to detect the protein levels of samples, which requires attention in future studies.

## Conflict of Interest

There was no conflict of interest.

## References

[R1] Acevedo-Rodriguez A, Kauffman A, Cherrington B, Borges C, Roepke TA, Laconi M (2018). Emerging insights into hypothalamic‐pituitary-gonadal axis regulation and
interaction with stress signalling. J Neuroendocrinol.

[R2] Chatterjee A, Rajikin MH, Chatterjee R, Ghosh S (2006). Stress and how it affects reproduction. Biomed Res.

[R3] Morales Ramírez, Vargas Estrada, Juárez Rodríguez, Pérez-Rivero JJ, Sierra Reséndiz, Flores González (2022). Effects of phytoestrogens on the reproductive physiology of productive
species Review. Rev Mex Cienc Pecu.

[R4] Szeliga A, Czyzyk A, Podfigurna A, Genazzani AR, Genazzani AD, Meczekalski B (2018). The role of kisspeptin/neurokinin B/dynorphin neurons in pathomechanism
of vasomotor symptoms in postmenopausal women: from physiology to potential
therapeutic applications. Gynecol Endocrinol.

[R5] Uenoyama Y, Nagae M, Tsuchida H, Inoue N, Tsukamura H (2021). Role of KNDy neurons expressing kisspeptin, neurokinin B, and dynorphin A
as a GnRH pulse generator controlling mammalian reproduction. Front Endocrinol.

[R6] Khazali H, Mahmoudi F, Janahmadi M (2018). Hypothalamic KiSS1/GPR54 gene expressions and luteinizing hormone plasma
secretion in morphine treated male rats. Int J Fertil Steril.

[R7] Clarke H, Dhillo WS, Jayasena CN (2015). Comprehensive review on kisspeptin and its role in reproductive
disorders. Endocrinol Metab.

[R8] Mahmoudi F, Khazali H, Janahmadi M (2014). Morphine attenuates testosterone response to central injection of
kisspeptin in male rats. Int J Fertil Steril.

[R9] Rance NE, Krajewski SJ, Smith MA, Cholanian M, Dacks PA (2010). Neurokinin B and the hypothalamic regulation of reproduction. Brain RES.

[R10] Anderson R, Skorupskaite K, Sassarini J (2019). The neurokinin B pathway in the treatment of menopausal hot flushes. Climacteric.

[R11] Young J, George JT, Tello JA, Francou B, Bouligand J, Guiochon-Mantel A (2013). Kisspeptin restores pulsatile LH secretion in patients with neurokinin B
signaling deficiencies: physiological, pathophysiological and therapeutic
implications. Neuroendocrinology.

[R12] Akbaribazm M, Khazaei F, Naseri L, Pazhouhi M, Zamanian M, Khazaei M (2021). Pharmacological and therapeutic properties of the Red Clover (Trifolium
pratense L) an overview of the new findings. J Tradit Chin Med.

[R13] Ding M, Bao Y, Liang H, Zhang X, Li B, Yang R (2024). Potential mechanisms of formononetin against inflammation and oxidative
stress: a review. Front Pharmacol.

[R14] Mu H, Bai Y-H, Wang S-T, Zhu Z-M, Zhang Y-W (2009). Research on antioxidant effects and estrogenic effect of formononetin
from Trifolium pratense (red clover). Phytomedicine.

[R15] Yigit E, Unsal S (2024). Isoflavones obtained from red clover improve both dyslipidemia and
menopausal symptoms in menopausal women: a prospective randomized placebo-controlled
trial. Climacteric.

[R16] Oza MJ, Kulkarni YA (2018). Formononetin treatment in type 2 diabetic rats reduces insulin resistance
and hyperglycemia. Front Pharmacol.

[R17] Wang X-s, Guan S-y, Liu A, Yue J, Hu L-n, Zhang K (2019). Anxiolytic effects of Formononetin in an inflammatory pain mouse model. Mol brain.

[R18] Ghazanfarpour M, Sadeghi R, Roudsari RL, Najmabadi KM, Khadivzadeh T (2015). Effects of red clover on hot flash and circulating hormone concentrations
in menopausal women: a systematic review and meta-analysis. Avicenna J Phytomed.

[R19] Paxinos G, Watson C (2006).

[R20] Basirat E, Mahmoudi F, Khazali H (2025). The expression of melanin concentrating hormone and corticotrophin
releasing hormone genes in a stress model rats receiving formononetin. Gene, Cell, Tissue.

[R21] McGarvey C, Cates PS, Brooks AN, Swanson IA, Milligan SR, Coen CW (2001). Phytoestrogens and gonadotropin-releasing hormone pulse generator
activity and pituitary luteinizing hormone release in the rat. Endocrinology.

[R22] Kelly MJ, Rønnekleiv OK (2015). Minireview: neural signaling of estradiol in the hypothalamus. Mol Endocrinol.

[R23] Azcoitia I, Mendez P, Garcia-Segura LM (2021). Aromatase in the human brain. Androg Clin Res Ther.

[R24] Korani M (2023). Aromatase inhibitors in male: A literature review. Med Clin Práctica.

[R25] Sandini TM, Reis-Silva TM, Moreira N, Bernardi MM, Lebrun I, Spinosa HdS (2019). Effects of isoflavones on behavior, estradiol, glutamate, and GABA levels
in intact middle-aged female rats. Nutr Neurosci.

[R26] Li S, Dang Y, Zhou X, Huang B, Huang X, Zhang Z (2015). Formononetin promotes angiogenesis through the estrogen receptor
alpha-enhanced ROCK pathway. Sci Rep.

[R27] Kuiper GG, Lemmen JG, Carlsson B, Corton JC, Safe SH, Van Der (1998). Interaction of estrogenic chemicals and phytoestrogens with estrogen
receptor β. Endocrinology.

[R28] Chen Y-M, Wang I-L, Zhu X-Y, Chiu W-C, Chiu Y-S (2021). Red clover isoflavones influence estradiol concentration, exercise
performance, and gut microbiota in female mice. Front Nutr.

[R29] Waris Gh, Ahmed A, Rukhsar A, Zahra F, A M (2023). Formononetin as promising therapeutic intervention for restoring ovarian,
uterine, and hepato renal functions in letrozole- induced polycystic ovarian
syndrome sprague dawley. J Popul Ther Clin Pharmacol.

[R30] Qiu J, Rivera HM, Bosch MA, Padilla SL, Stincic TL, Palmiter RD (2018). Estrogenic-dependent glutamatergic neurotransmission from kisspeptin
neurons governs feeding circuits in females. Elife.

[R31] Nestor CC, Qiu J, Padilla SL, Zhang C, Bosch MA, Fan W (2016). Optogenetic stimulation of arcuate nucleus Kiss1 neurons reveals a
steroid-dependent glutamatergic input to POMC and AgRP neurons in male mice. Mol Endocrinol.

[R32] Iwata K, Ogata R, Sato M, Matsuda F, Ishii H, Ozawa H (2023). Short-term depletion of plasma estrogen affects hypothalamic
kisspeptin-neurokinin B-dynorphin a neurons, gonadotrophs, and pulsatile luteinizing
hormone secretion in female rats. Peptides.

[R33] Morris PG, Herbison AE (2023). Mechanism of arcuate kisspeptin neuron synchronization in acute brain
slices from female mice. Endocrinology.

[R34] Cavendish RL, de Souza, Neto RB, Paixão AO, Oliveira JV, de Araujo (2015). Antinociceptive and anti-inflammatory effects of Brazilian red propolis
extract and formononetin in rodents. J Ethnopharmacol.

[R35] Tian J, Wang X-Q, Tian Z (2022). Focusing on formononetin: recent perspectives for its neuroprotective
potentials. Front Pharmacol.

[R36] Yu D, Duan Y, Bao Y, Wei C, An L (2005). Isoflavonoids from Astragalus mongholicus protect PC12 cells from
toxicity induced by L-glutamate. J Ethnopharmacol.

[R37] Prague JK, Voliotis M, Clarke S, Comninos AN, Abbara A, Jayasena CN (2019). Determining the relationship between hot flushes and LH Pulses in
menopausal women using mathematical modeling. J Clin Endocrinol Metab.

[R38] Jayasena CN, Comninos AN, Stefanopoulou E, Buckley A, Narayanaswamy S, Izzi-Engbeaya C (2015). Neurokinin B administration induces hot flushes in women. Sci Rep.

